# Automatic digital quantification of bone marrow myeloma volume in appendicular skeletons - clinical implications and prognostic significance

**DOI:** 10.1038/s41598-017-13255-w

**Published:** 2017-10-10

**Authors:** Yuki Nishida, Shinya Kimura, Hideaki Mizobe, Junta Yamamichi, Kensuke Kojima, Atsushi Kawaguchi, Manabu Fujisawa, Kosei Matsue

**Affiliations:** 1 Division of Hematology, Respiratory Medicine and Oncology, Department of Internal Medicine, Faculty of Medicine, Saga University, Saga, Japan; 2 Medical Equipment Business Planning Department, Medical Equipment Group, Canon Inc., Kawasaki, Japan; 3 Section of Clinical Cooperation System, Center for Comprehensive Community Medicine, Faculty of Medicine, Saga University, Saga, Japan; 4 Division of Hematology/Oncology, Department of Medicine, Kameda Medical Center, Kamogawa, Japan

## Abstract

Multiple myeloma (MM) is a clonal plasma cell disorder originating in bone marrow. Whole body low-dose multidetector CT (MDCT) can depict bone marrow infiltration by myeloma cells into the adipose-rich fatty marrow of the appendicular skeleton. However, automated and objective volume measurement of bone marrow infiltration has not been established, and its clinical relevance remains unclear. We therefore developed novel CT post-processing software (MABLE software) and measured the total sum of CT values (cumulative CT value, cCTv) representing bone marrow infiltration, by combining volume and voxel-based CT values. The cCTv was greater in patients with symptomatic MM than in those with smouldering MM or monoclonal gammopathy of unknown significance. Patients with revised International Staging System (R-ISS) III had a higher cCTv than those with R-ISS I or II. Age, albumin, and M-protein levels independently predicted cCTv. Mixed graphical model analysis revealed direct relationships between cCTv and age or R-ISS. Tree-structured survival analysis and multivariate Cox analysis revealed that a cCTv greater than or equal to 4.4 was independently prognostic for overall survival. Anti-myeloma therapy reduced cCTv after treatment. These findings suggest that the automatically calculated cCTv reflects disease aggressiveness and is useful for accurate prognostic prediction in MM patients.

## Introduction

Multiple myeloma (MM) is a disease of malignant plasma cells in the bone marrow and is characterised by anaemia, lytic bone lesions, renal disorder, and hypercalcaemia^1^. Although novel therapies have markedly improved the survival of patients with MM, MM remains an incurable disease. The international staging system (ISS) is a simple laboratory test-based risk stratification algorithm for predicting the survival of newly-diagnosed MM patients^2^. Specific recurrent chromosomal abnormalities (CA) and serum lactate dehydrogenase (LDH) have been found to be useful as prognostic markers^3–9^, and their incorporation into the ISS as the Revised ISS (R-ISS) has improved the predictive value for MM^10–12^.

The extent of bone marrow infiltration by myeloma cells, as evaluated by bone marrow biopsy, is one of the important determinants of the myeloma tumour burden; however, assessing the volume of myeloma infiltration in bone marrow is difficult, because of the patchy distribution of bone marrow plasma cells. Whole body low-dose multidetector CT (MDCT) is a widely used noninvasive and low time-consuming imaging technique, and is accepted for the initial workup of symptomatic MM in the current consensus statement from the International Myeloma Working Group (IMWG)^13,14^. MDCT can clearly depict bone marrow infiltration in the adipocyte-rich tissue of long bones (i.e., the humeri and femora), where it is observable as hyper attenuating lesions^15,16^.

We previously reported that CT values [also known as Hounsfield units (HUs) scale] in the long bones reflected disease stages in plasma cell dyscrasia, with higher CT values predicting inferior prognoses^17^. However, the previous method for determining CT values in the bones lacked objectivity, as the region-of-interest was selected by each inspector. To quantify the exact volume of the bone marrow lesions and their impacts on the survival of MM patients, and also to establish a method that withstands use in global clinical studies, we developed a novel convenient and objective method using Lesion Management Solutions (LMS) developed by MEDIAN Technologies^18^.

In the present retrospective study, we used MDCT to determine the total CT values of abnormal bone marrow infiltration (named the cumulative CT value, cCTv) in the femora and humeri of newly-diagnosed MM patients. This was accomplished by combining the voxel CT values and myeloma tumour volume using automated post-processing software (named MyelomA Bone marrow Lesion: MABLE). We then explored the clinical relevance of cCTv in respect to demographic and laboratory variables.

## Patients and Methods

### Patients

Between January, 2008, and December, 2015, 91 patients with newly-diagnosed symptomatic MM were examined with MDCT at Kameda Medical Center. Patients with histologically proven symptomatic MM were enrolled in the study. Patients with previous or current histories of malignancies other than myeloma, causes of anaemia other than myeloma, and histories of orthopaedic surgery to the appendicular skeleton were excluded. Twenty-seven patients with smouldering MM (SMM) and nine patients with monoclonal gammopathy of unknown significance (MGUS) who received MDCT for initial investigations of bone lesions were included for comparison purposes. Follow-up MDCT assessment was performed in 32 patients with symptomatic MM. Written informed consent was obtained from each patient, and this study was approved by the institutional review board of Kameda Medical Center (09–014), in accordance with the Declaration of Helsinki 1975, as revised in 2008.

### Initial Workup for myeloma and treatment information

Diagnoses of MM and MGUS were made according to the International Myeloma Working Group (IMWG) criteria^14^. The workup of MM included serum and urine immunofixation electrophoresis, quantitation of serum immunoglobulin and free light chain (κ, λ and κ/λ ratio), 24-hour urinary protein excretion, haemoglobin, serum albumin, beta-2-microglobulin (β_2_MG), and LDH levels. Bone marrow aspiration and biopsy from the unilateral iliac crest were performed. Fluorescence *in situ* hybridization analyses were performed according to the standard method. High-risk CAs were defined as t(4;14), t(4;16), and 17p deletions detected by Fluorescence *in situ* hybridization^19^. High LDH was defined as a serum level greater than the upper limit of the normal range. MM patients were re-classified under the criteria for R-ISS^11^. All of the patients were treated with bortezomib-based regimens as the first-line therapy. Suitable patients received high-dose melphalan plus autologous stem cell transplantation (HDM + ASCT). Treatment responses were defined according to IMWG response criteria.

### Imaging acquisition and cCTv calculation by the MABLE post-processing software

All CT examinations were performed at Kameda Medical Center. The image acquisition by MDCT (120 kV; 80 mA) has been described previously^17^. After acquisition, the CT images were processed using the MABLE software, which automatically calculated the sum of voxel CT values. The ranges of CT values for bone (over 120 HU), bone marrow (−200 to −30 HU), and myeloma (−30 to 120 HU) were determined, as the normal bone marrow in the long bones of adults is rich in adipocytes, and may be represented by CT values of up to −30 HU, according to a previous report^16^.

**Figure 1:**
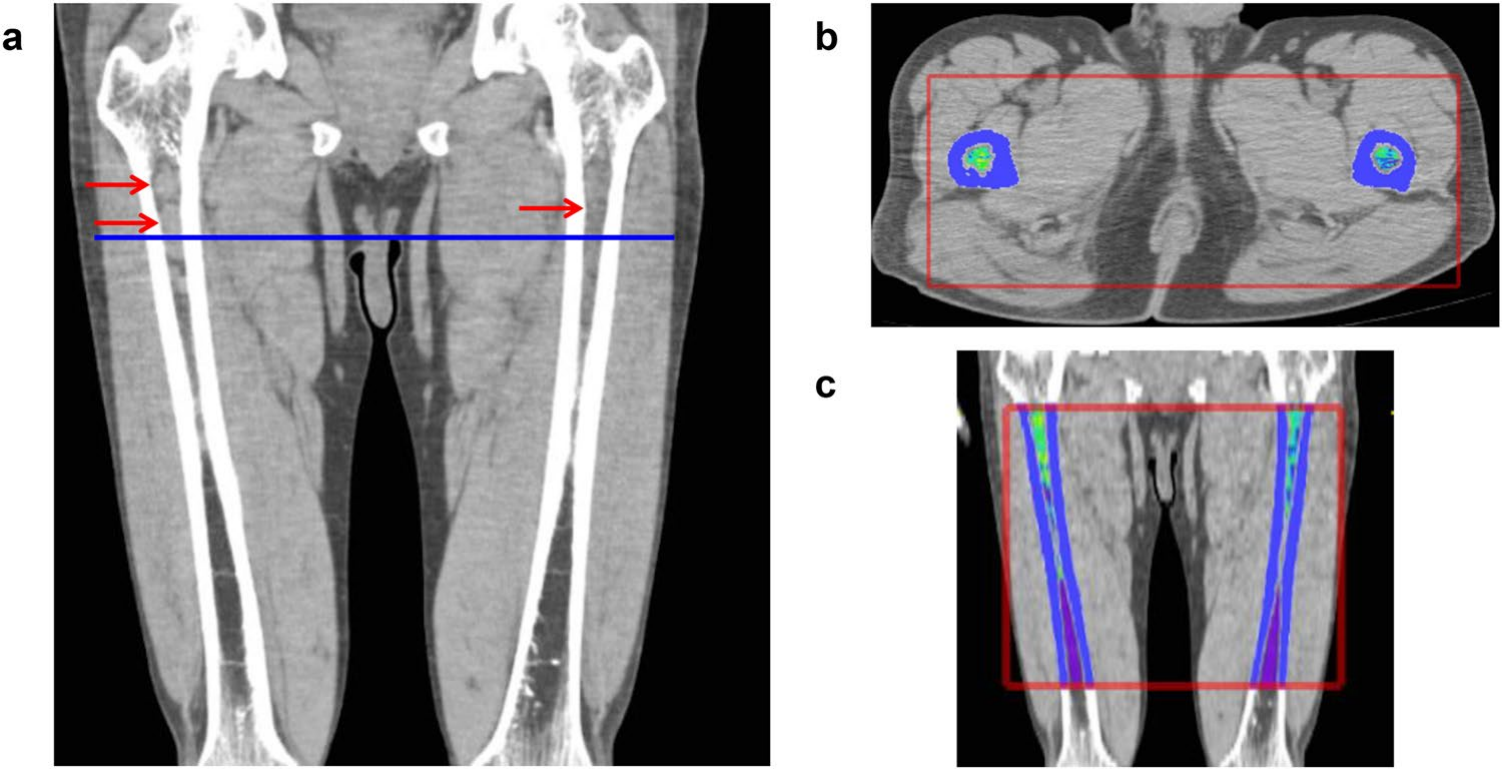
Representative images of a patient with advanced IgAκ myeloma. (**a**) Coronal CT image of the femora. Myeloma infiltration was detected in the upper metaphysis and diaphysis in both the left and right femora (arrows). (**b**) Axial image at the level of the blue line shown in (**a**). The software provides pseudo-colour according to the Hounsfield unit (HU) (high: yellow-red, low: blue-purple). (**c**) Coronal image reconstructed from the axial slices. Red rectangle: horizontal and vertical ranges for the determination of cumulative CT value (cCTv). The cCTv of this case was 4.46 HU.

To determine a three-dimensional volume-of-interest and calculate the sum of CT values in one appendicular skeleton, the software set horizontal and vertical ranges on axial CT images. The horizontal range (x and y-axis) (e.g., shown as the red rectangle in Fig. 1b) included a bone marrow area surrounded by circular cortical bone. The software automatically recognized circular regions with CT values higher than 120 HU as cortical tubular bones. The vertical range (z-axis) (e.g., shown as the red rectangle in Fig. 1c) was anatomically defined by the operators to include the diaphysis and proximal and distal metaphysis and to exclude the proximal and distal epiphysis, as the trabecular architecture of the epiphysis expresses very high (over 120 HU) CT values. Once horizontal and vertical ranges in each appendicular skeleton had been selected, the software automatically extrapolated the ranges rest of the slices, and calculated cCTv for all the voxels within the selected bone.

To offset anatomical variations in each bone and patient, we took the products determined by the voxel HU and myeloma volume and divided them by the volume of the bone marrow cavity consisting of normal bone marrow and myeloma infiltration. The formula for the calculation of an appendicular skeleton was as follows: Value of abnormal bone marrow lesion (HU) = cumulative voxel HU of myeloma × total volume of myeloma (mm^3^) / total volume of bone marrow cavity (normal bone marrow + myeloma infiltration) (mm^3^).

To offset negative values, the program automatically added 30 HU to each measured voxel included as myeloma, and regarded voxels included as fatty marrow with values of −30 HU or lower as 0 HU. As the voxel sizes of the CT images of each patient may have differed, the program applied a resampling function and reconstructed all original CT images to a 1-mm cubic voxel resolution. We took the logarithm (log_10_ scale) of the sum of the values calculated on two humeri and femora in each patient, and termed this logarithmic value the cCTv. The differences in cCTv before and after treatment were calculated for the patients who received follow-up MDCT according to the formula: [the raw sum of CT values (before)] – [the raw sum of CT values (after)], with the values being expressed logarithmically.

### Statistical analysis

For comparisons between two groups, chi-square tests were performed on categorical variables, and Wilcoxon rank-sum tests on continuous variables. One-way ANOVA with Bonferroni correction was performed for comparisons between three groups. Correlations between cCTv and staging systems (Durie-Salmon [D-S] stage, ISS, and R-ISS) were calculated using Pearson’s correlation coefficient. Multiple linear regression analysis was used to investigate clinical parameters associated with cCTv. Overall survival was defined as the date of diagnosis until death from any cause; survivors were censored at the time of the last contact or the end of October, 2016. The Kaplan-Meier method was used to estimate the median overall survival and comparisons were performed using the log-rank test.

A mixed graphical model based on the methodology of Edwards *et al*.^20^ was used to describe correlations between clinical variables. Tree-structured survival analysis was used to identify the greatest differences in survival curves by dividing the patients into the most appropriate groups according to the parameters of interest^21^.

Data analysis was performed with Stata SE software version 13.1 (Stata Corp., College Station, TX, USA) and R statistical software version 3.3.2 (R Foundation for statistical Computing, Vienna, Austria). All statistical test values were two-sided, and *P* values of < 0.05 were taken to indicate statistical significance in all analyses.

## Results

### High cCTv indicates high tumour burden and aggressive disease, as defined by the R-ISS

In total, the software was used to perform post-processing analysis in 127 patients with MGUS, SMM, and symptomatic MM (left and right humeri and femora, resulting in 508 appendicular skeletons). The minimum voxel CT values of normal bone marrow ranged from −200 to −105 HU (98% of examined voxels were ≥ −199 HU), while the maximum voxel CT values of abnormal plasma cell infiltrations ranged from −30 to 120 HU (98% of examined voxels were ≤ 119 HU), indicating that the assigned ranges for these tissues were appropriate, and included the distribution of voxel CT values of bone marrow and myeloma tissue in patients with plasma cell dyscrasia.

We observed two patients whose bone marrow lesions detected by MDCT were histologically proven as malignant plasma cell infiltrations (Figure S1). Figure 1 shows an example of femur images from a patient with IgA myeloma of D-S stage III, ISS stage III, and R-ISS stage III, which resulted in a high cCTv measurement by the software. The characteristics of patients with symptomatic MM are summarised in Table 1. The median follow-up duration was 30.2 months, the median age was 73 years (range: 44 to 89 years), and 49 (54%) patients were male. Of the total 91 patients, 50 had IgG myeloma, 27 had IgA myeloma, 13 had Bence-Jones myeloma, and one had non-secretory myeloma. Sixty-two (69%) patients had D-S stage III and 43 (47%) patients had ISS stage III. Overall, 19 (21%) patients had high-risk CAs [12 harboured t(4;14), 6 harboured del(17p), and 1 harboured t(14;16)] and 14 (15%) patients had elevated serum LDH levels. Thirty (33%) patients received HDM + ASCT, while 26 (28%) patients died during the follow-up period.

**Table 1:** Baseline patient characteristics.

	(range or %)
Median age	73 (44–89)
Male sex	49 (54)
IgG subtype	50 (55)
D-S stage III	63 (69)
ISS III	43 (47)
High-risk CA	19 (21)
High LDH	14 (15)
Revised ISS III	21 (23)
HDM + ASCT	30 (33)

Abbreviations: IgG, immunoglobulin G; D-S, Durie-Salmon; ISS, International Staging System; CA, chromosomal abnormalities; LDH, lactate dehydrogenase; HDM, high-dose melphalan; ASCT, autologous stem cell transplantation.

**Figure 2:**
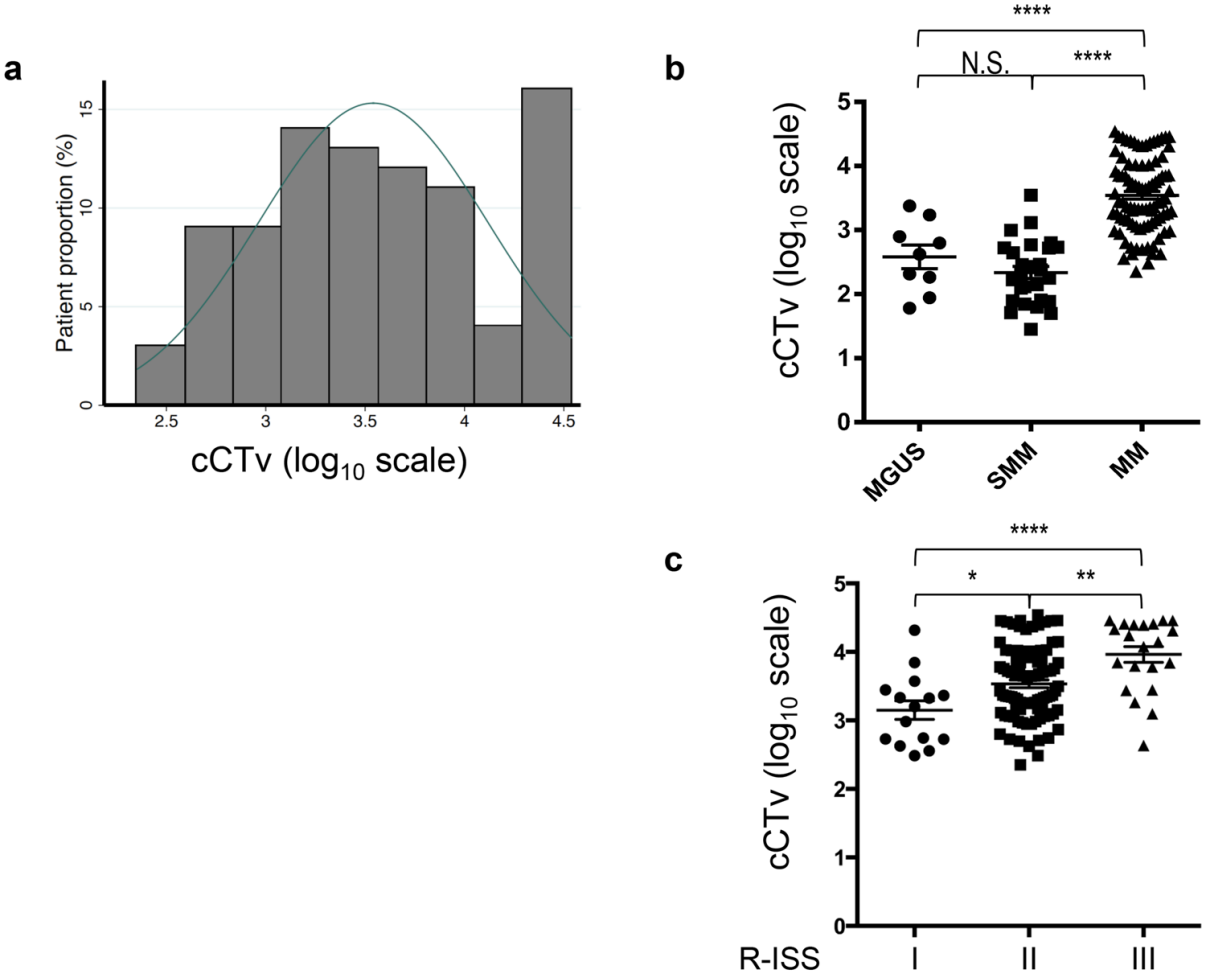
(**a**) Distribution of cumulative CT values (cCTv) in patients with MM. (**b**) Levels of cCTv in MGUS patients and MM patients. (**c**) Levels of cCTv in patients with MM grouped according to the revised international staging system. *P < 0.05, **P < 0.01, ****P < 0.0001 by one-way ANOVA with Bonferroni correction. Error bars denote SEM. MM, multiple myeloma; SMM, smouldering MM; MGUS, monoclonal gammopathy of unknown significance, N.S., not significant.

The raw values of abnormal bone marrow lesions in patients with symptomatic MM showed a right-skewed distribution, with over 60% of patients being included in the lowest group (Figure S2). After performing logarithmic transform of the values, cCTv varied across the patients and seemed to distribute normally, although a group of patients with a cCTv of 4.5 did not follow a normal distribution (Fig. 2a). This group consisted of 14 patients, 8 of whom (57%) died during the follow-up period, raising the possibility that a high cCTv could reflect high mortality in patients with symptomatic MM. To investigate whether cCTv correlated with disease stage in plasma cell dyscrasia, we compared the cCTvs in patients with symptomatic MM with those from patients with SMM and MGUS. The mean cCTv was higher in the symptomatic MM patients than in the SMM or MGUS patients (mean ± SEM: 3.55 ± 0.06 vs 2.34 ± 0.1 and 3.55 ± 0.06 vs 2.58 ± 0.18, *P* < 0.0001 by one-way ANOVA with Bonferroni correction; Fig. 2b). Among the symptomatic MM patients, the cCTv significantly correlated with D-S stage, ISS stage, and R-ISS stage (*r* = 0.39, *P* = 0.0007; *r* = 0.39, *P* = 0.0007 and *r* = 0.45, *P* < 0.0001, respectively). The mean cCTv in patients with R-ISS stage III was significantly higher than that in patients with R-ISS stage I & II (mean ± SEM: 3.96 ± 0.11, 3.49 ± 0.07, and 3.15 ± 0.14 in patients with R-ISS stage III, R-ISS stage II and R-ISS stage I respectively; *P* < 0.0001 by one-way ANOVA with Bonferroni correction; Fig. 2c), indicating that a high cCTv reflects advanced and aggressive features of myeloma. The median cCTv of patients with symptomatic MM was 3.45. Three patients with R-ISS I had a cCTv higher than the median, while five patients with R-ISS III had a cCTv lower than the median. The former three patients had a younger age than the mdian (44, 71 and 62 years) and D-S stage III, with two of them having received HDM + ASCT. All three patients were alive at the end of the observation period. With the exception of one patient, the latter group of five patients with R-ISS III had a relatively older age (71, 75, 88, 52 and 80 years). Two of these patients had cytogenetic abnormalities with a poor prognosis (17p deletion and IgH-FGFR3 fusion). Only one patient received HDM + ASCT, and two patients died during the observation period.

To investigate the clinical parameters correlating with cCTv levels independently, we performed linear regression analysis on the clinical variables, including age, sex, haemoglobin, β_2_MG, serum albumin, high-risk CA, high LDH, and serum or urine M-protein levels. We found that age and albumin negatively impacted on cCTv (*t-*score −2.88 and *P* value 0.005, and *t*-score −2.15 and *P* value 0.035, respectively), and that M-protein levels positively correlated with cCTv (*t*-score 2.53 and *P* value 0.013; Table 2).

**Table 2:** Linear regression analysis for cumulative CT values and clinical parameters.

	*t* score	*P* value	95% CI
Age	−2.88	0.005	−0.026, −0.005
Male sex	0.22	0.829	−0.184, 0.229
Haemoglobin	−1.25	0.215	−0.087, 0.020
Serum albumin	−2.15	0.035	−0.306, −0.117
Serum β_2_-microglobulin	1.99	0.050	4.52 × 10^−6^, 0.179
High-risk CA	0.99	0.326	−0.133, 0.397
High LDH	−0.73	0.465	−0.455, −0.210
M-protein levels	2.53	0.013	9.03 × 10^-6^, 0.0007

Abbreviations: CI, confidence interval; CA, chromosomal abnormalities.

### cCTv shows a direct relationship with age and R-ISS, and high cCTv is an unfavourable prognostic factor in patients with MM

**Figure 3:**
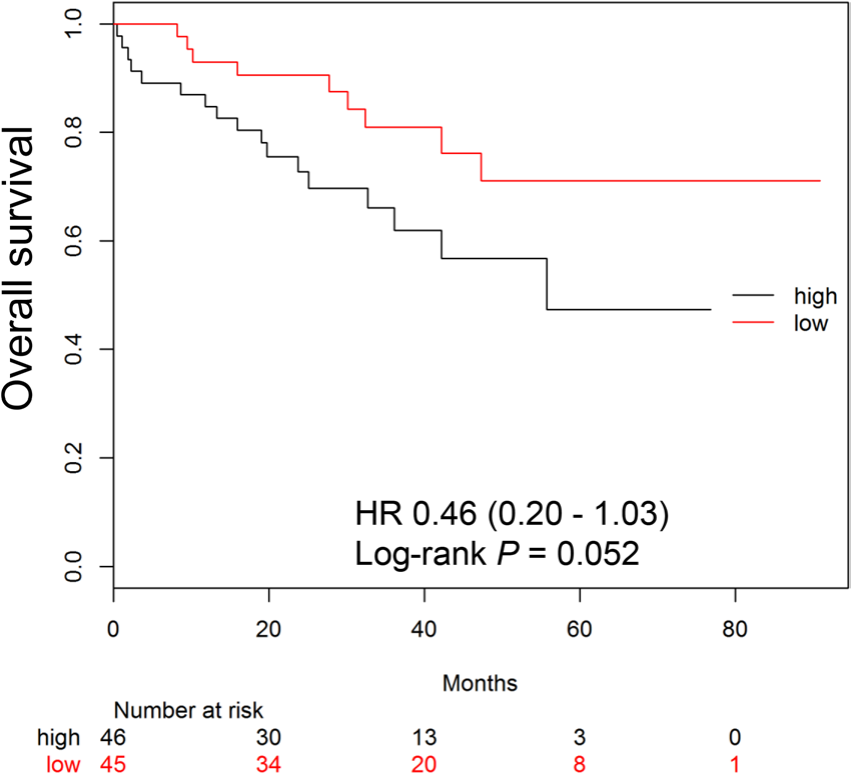
Kaplan-Meier survival curve for overall survival according to cumulative CT value (cCTv). Patients with multiple myeloma are dichotomised to high and low groups according to the median cCTv. HR, Hazard ratio.

To investigate the prognostic impact of cCTv, we next dichotomised patients into those with a cCTv above or equal to the median (high cCTv group), and those with values below the median (low cCTv group; Supplemental table). As expected, the number of patients with D-S stage III and ISS stage III was significantly higher in the high cCTv group than in the low cCTv group. The number of patients with high-risk CA and high LDH tended to be greater in the high cCTv group than in the low cCTv group, and the number of patients with R-ISS III was also significantly greater in the high cCTv group. A log-rank test showed that median overall survival in the high cCTv group was shorter than in the low cCTv group (not reached vs 55.7 months, hazard ratio 0.46, *P* = 0.052; Fig. 3).

**Figure 4:**
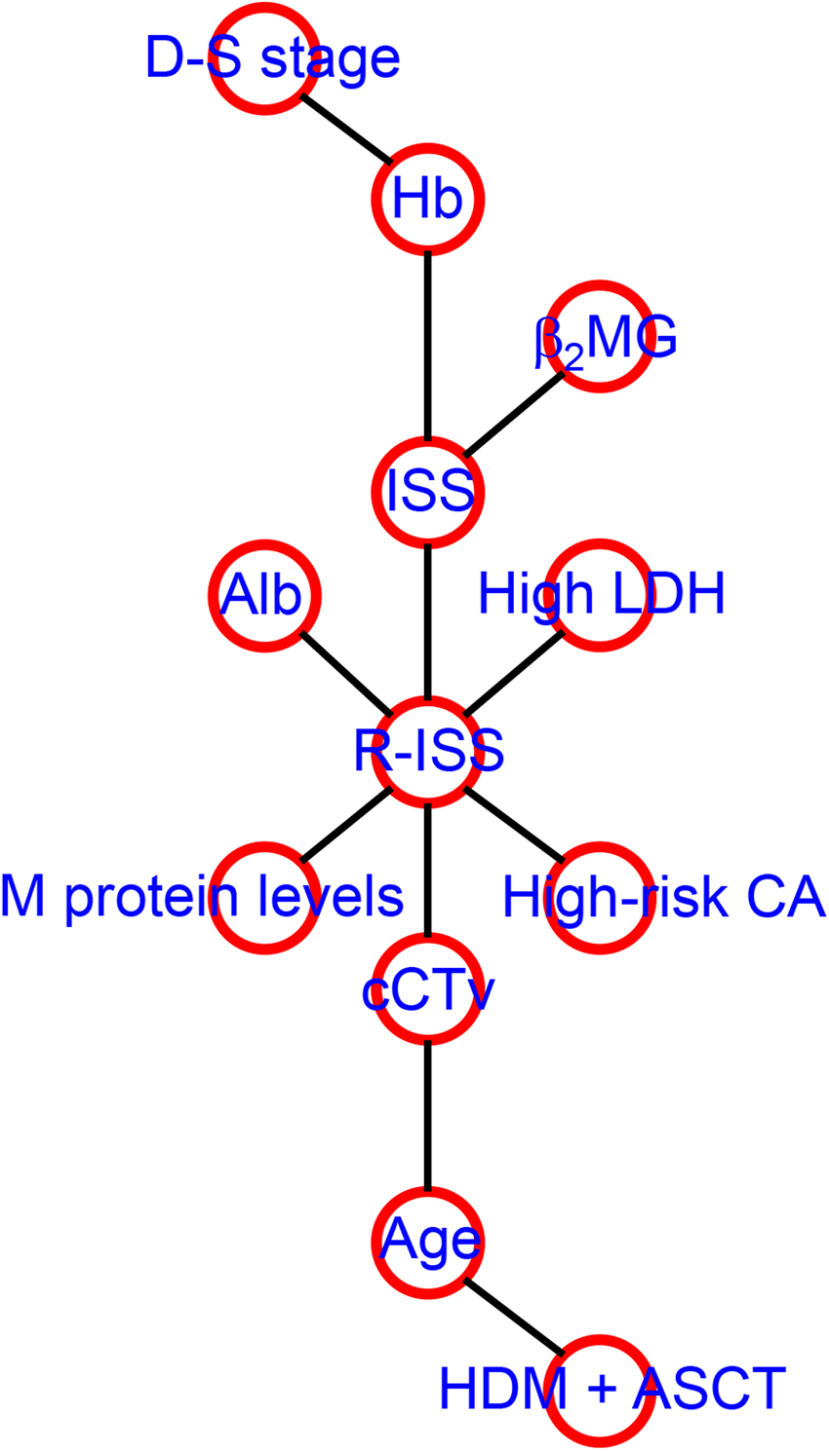
A graphic model of cCTv and clinical parameters in patients with MM as predicted by a mixed graphical model analysis. The network was visualised using R statistical software. D-S, Durie-Salmon; Hb, haemoglobin; β_2_MG, serum beta-2-microglobulin; ISS, international staging system; Alb, serum albumin; LDH, lactate dehydrogenase; R-ISS, revised international staging system; CA, chromosomal abnormalities; cCTv, cumulative CT values; HDM + ASCT, high-dose melphalan followed by autologous stem cell transplantation.

To further investigate relationships between the clinical variables and cCTv, we performed a mixed graphical model (MGM) analysis. This MGM analysis (depicted in Fig. 4) revealed that cCTv had direct relationships with both age and R-ISS.

As the patients in the present study were older than those in other clinical trials, and as age had a negative impact on cCTv according to the linear regression analysis, age may have attenuated the impact of cCTv on survival. Accordingly, we selected age, cCTv, and R-ISS as covariates, and performed tree-structured survival analysis to identify the greatest differences in survival curves by dividing patients into the most appropriate two groups according to these three parameters.

**Figure 5:**
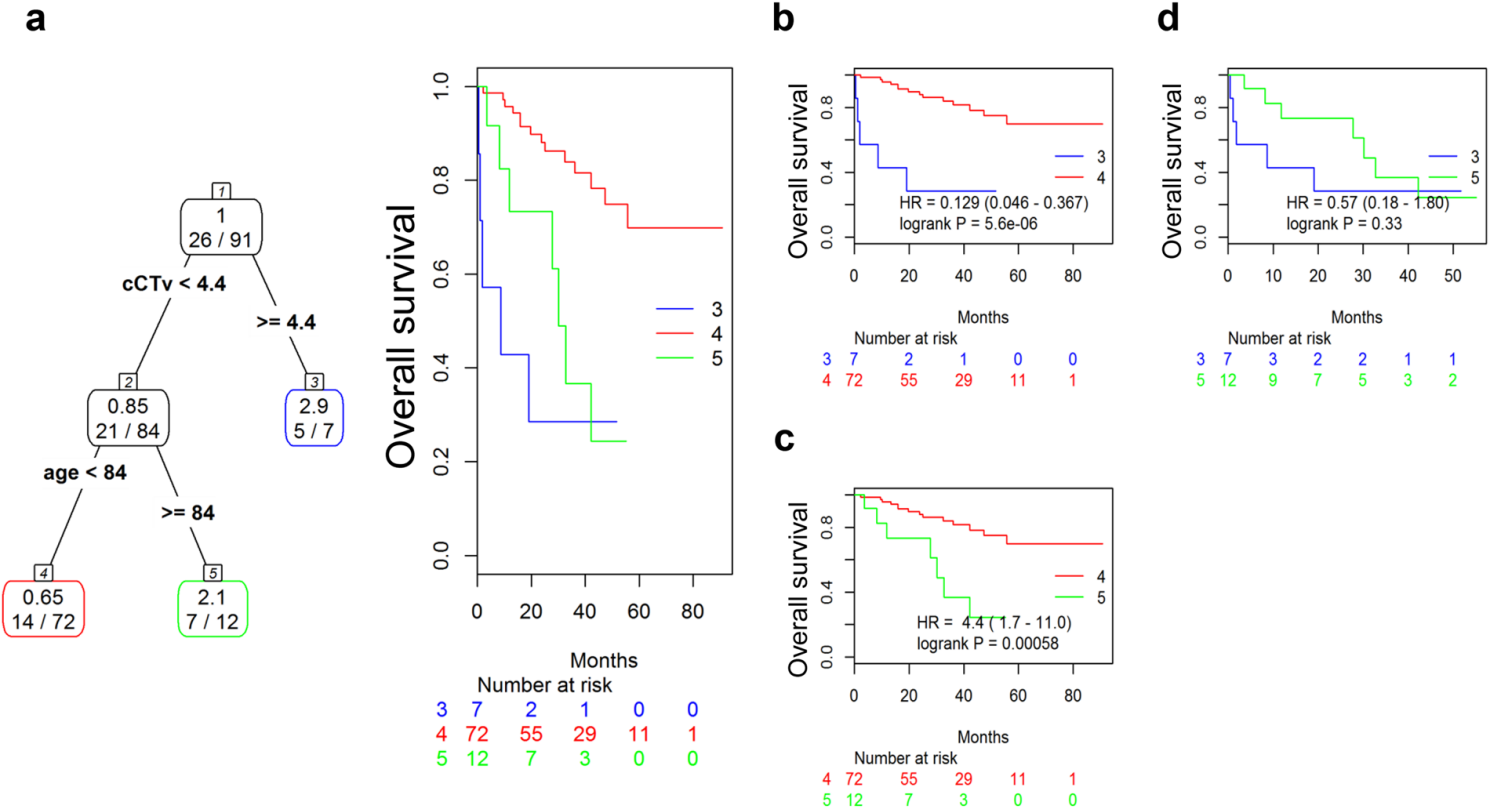
Patient stratification for overall survival by tree-structured survival analysis. (**a**) Tree-structured classification of patients according to cCTv and age (left panel). The numbers in the boxes represent relative risk of death (upper) and the numbers of death events/patients (lower). Kaplan-Meier curves are coloured according to the classification (right). Blue: Group 3, red: Group 4, and green: Group 5. (**b**–**d**) Kaplan-Meier curves and survival comparisons by log-rank test between the two groups stratified in (**a**). cCTv, cumulative CT value, HR, hazard ratio.

The tree-structured survival analysis identified a cCTv greater than or equal to 4.4 as the first split point (Fig. 5a). This divided the patients into a group of 7 (Group 3) and a group of 84. Patients with a cCTv less than 4.4 were further subdivided most appropriately by age, resulting in two subgroups, namely, patients with an age greater than or equal to 84 years (12 patients, Group 5), and those under 84 years (72 patients, Group 4). The Kaplan-Meier curves for overall survival of patients in Groups 3, 4, and 5 are presented in Fig. 5b–d. Log-rank tests revealed that patients in Groups 3 and 5 had significantly inferior overall survival compared with those in Group 4 [median survival, not reached vs. 8.6 months, hazard ration (HR) 0.129, *P* = 5.6 × 10^−6^ for Group 3 vs Group 4 and not reached vs. 30.1 months, HR 4.4, *P* = 0.00058 for Group 4 vs Group 5, respectively], while there was no significant difference in overall survival between the patients in Groups 3 and 5.

To further investigate whether these three groups were independent prognostic factors for survival, we performed a multivariate Cox analysis that included classification as Group 3 or 5 against Group 4, and other clinical parameters such as sex, D-S staging, R-ISS, and the presence of HDM + ASCT. The multivariate Cox analysis (Table 3) revealed that the presence of HDM + ASCT was an independent favourable prognostic factor (HR 0.26, *P* = 0.026), and that being in Group 3 or 5 was an independent unfavourable prognostic factor (HR 6.09, *P* < 0.001 and HR 5.08, *P* = 0.002, respectively). We performed a power analysis to examine the power in each subgroup shown in Fig. 5. The calculated powers were 97.2%, 86.1% and 23.1% for Fig. 5b,c and d, indicating that the numbers of patients had a sufficient power to distinguish patients with a cCTv of greater than or equal to 4.4 and a very poor outcome.

**Table 3:** Multivariate Cox analysis for overall survival.

	HR	Std. Err.	z	*P* value	95% CI
Male sex	1.92	0.421	1.55	0.121	0.84, 4.38
D-S stage	2.08	0.373	1.96	0.05	1.00, 4.32
R-ISS	1.64	0.396	1.25	0.212	0.75, 3.56
HDM + ASCT	0.26	0.608	−2.23	0.026	0.08, 0.85
Gr 3 (vs Gr 4)	6.09	0.549	3.29	<0.001	2.08, 17.85
Gr 5 (vs Gr 4)	5.08	0.513	3.16	0.002	1.86, 13.88

Abbreviations: D-S, Durie-Salmon; R-ISS, revised international staging system; HDM, high-dose melphalan; ASCT, autologous stem cell transplantation; Gr, group; HR, hazard ratio; Std. Err., standard error; CI, confidence interval.

### Anti-myeloma therapy reduces cCTv

We compared cCTv before and after myeloma treatments. Up until the end of the follow-up, 32 patients received an MDCT assessment after the treatment. The median duration between initial and follow-up MDCT assessments was 365 days. Of the 32 patients, two achieved a complete response (CR), 11 achieved a stringent CR, seven achieved an immunophenotypic CR, five achieved a very good partial response (VGPR), and seven achieved a partial response (PR). Overall, cCTv was significantly lower after the treatments than before the treatments (mean ± SEM, 3.20 ± 0.08 vs 3.70 ± 0.11, *P* < 0.0001). The differences in cCTv before and after treatment did not change according to treatment response (CR, VGPR, and PR; *P* = 0.9477 by one-way ANOVA; Figure S3a). We performed further univariate logistic regression analysis on the differences in cCTv according to whether patients achieved a CR or not. Differences in cCTv were not significantly influenced by the achievement of a CR (odds ratio: 1.00, *P* = 0.733). However, among the patients who achieved a CR, we found that those who achieved an immunophenotypic CR had a significantly greater pre to post treatment difference in cCTv than those who achieved a stringent CR or CR (*P* = 0.007; Figure S3b).

## Discussion

MDCT has been utilised for the diagnosis and staging of MM patients^22–26^. In particular, bone marrow infiltration of the long bones has been recently investigated, with the diameters of at least two medullary lesions having been utilised for response monitoring^27^. A computer-based approach for the automated detection of bone marrow infiltration has also been validated^28^. As we showed in Figure S1, MDCT was able to detect bone marrow lesions that could lead to unexpected fractures, supporting the idea that focusing on bone marrow infiltration is clinically useful. However, the volume of myeloma has not been intensively investigated. One study using ^18^F-FDG PET/CT demonstrated that calculating the volume of myeloma with a maximum standard uptake value (SUV_max_) of greater than 2.5 allowed patients with a worse prognosis to be distinguished^29^. However, the volume of myeloma infiltrating the bone marrow has not been directly measured and evaluated as a target for assessing disease burden. Our MABLE software was able to automatically distinguish bone marrow tissue from compact bone according to their substantial differences in CT value (−200 to −30 HU vs 400 to 1000 HU, respectively), thereby resulting in the elimination of subjectivity.

We found that cCTv had significant positive correlations with serum or urine M-protein levels, and a negative correlation with serum albumin (Table 2). We observed discrepancies in some patients who had R-ISS I and higher cCTv levels than the median, and in others with R-ISS III and lower cCTv levels than the median (three and five patients, respectively). These could be explained by the association of age and M-protein levels, which had significantly negative and positive impacts on cCTv respectively; i.e., those with a younger age and high M-protein levels had relatively higher cCTv levels than other patients in R-ISS III, while those with an older age and low M-protein levels had lower cCTv levels than other patients in R-ISS III. cCTv correlated more strongly with R-ISS than with D-S stage or ISS. The MGM revealed a direct relationship between cCTv and R-ISS (Fig. 4). Patients with a cCTv higher than the median value tended to have high serum LDH, high-risk of CA, a greater proportion of R-ISS III (Supplemental table), and worse survival (Fig. 3) than those below the median value. These findings indicate that patients with a high cCTv tend to have biologically aggressive MM. As R-ISS is a novel prognostic stratification, there is currently no study that has investigated the relationship between imaging results and R-ISS.

We found that age was a significant negative determinant for cCTv (Table 2) and had a direct relationship with cCTv according to the MGM (Fig. 4). MRI-based studies show that the fat content in the bone marrow of vertebrae and femurs gradually increases with advancing age, while the water content gradually decreases^30–32^. Another PET study shows that the SUV_max_ in proximal femoral and humeral red marrow significantly decreases with advancing age^33^. As water has a higher CT value than fat, these findings support our linear regression results indicating that age negatively impacted on cCTv. These findings may also support the idea that age attenuated the impact of cCTv on survival. Nevertheless, the distribution of cCTv showed a distinct population with a high cCTv of 4.5 and high mortality (57%), while the tree-structured survival analysis, a more sophisticated way to discriminate subgroups in terms of survival, primarily identified a group of patients with a cCTv greater than or equal to 4.4 (Group 4 in Fig. 5a). Five patients out of 7 (71%) died during the follow-up period, with a median survival of only 8.6 months. The multivariate Cox analysis showed that a cCTv greater than or equal to 4.4 was an independent unfavourable prognostic factor (Fig. 5 and Table 3), raising the possibility that measurement of cCTv can identify a subset of patients with a high volume of bone marrow infiltration represented by high voxel CT values, and with very aggressive disease and a poor outcome. Although the proportion of such patients was relatively small (8%), a similar proportion of patients (10%) with inferior survival has been identified by a scoring system using a combination of ^18^F-FDG PET/CT results and treatment response^34^.

MDCT is advantageous for the initial diagnostic workup in MM patients, as MDCT can be performed in a very short time (around 45 seconds) and is used worldwide. According to the OECD database, CT is a more commonly used modality than MRI or ^18^F-FDG PET/CT^35^. MDCT is a lower-cost- and less time-consuming imaging technique than whole body MRI or ^18^F-FDG PET/CT. Our current approach can be performed globally, as the MABLE software can be applied to all generally used CT scanners. With consideration of the fact that the software does not require the skills of experienced radiologists, clinical trials using the approach for initial diagnostic investigations and response assessment could be performed globally. As an example of such a software approach, a cloud-based image evaluation study has already been proposed as a tool for global clinical trials in oncology^18^.

In conclusion, we found that cCTv demonstrated a relationship with disease aggressiveness and had prognostic value as a measure of total bone marrow infiltration in the appendicular skeleton of MM patients. Moreover, cCTv can be automatically computed from MDCT images by MABLE software. A cCTv cutoff above or equal to 4.4 identified a subgroup of patients (8%) with very poor outcomes, who may require alternative treatment strategies. These findings suggest that automatic calculation of cCTv could be suitable for global studies, because of its objectivity and convenience, and that it could improve the accuracy of prognostic predictions for MM patients.

## Electronic supplementary material

Supplementary Information
