# Supplementary material for: Automatic digital quantification of bone marrow myeloma volume in appendicular skeletons - clinical implications and prognostic significance
Source: Sci Rep. 2017 Oct 10;7:12885. doi: 10.1038/s41598-017-13255-w (PMC5635114; doi:10.1038/s41598-017-13255-w)
Supplement: Supplementary file 1 — Supplementary Information [file 41598_2017_13255_MOESM1_ESM.pdf]

Automatic digital quantification of bone marrow myeloma volume in appendicular skeletons - clinical implications and prognostic significance

Yuki Nishida, Shinya Kimura, Hideaki Mizobe, Junta Yamamichi, Kensuke Kojima, Atsushi Kawaguchi, Manabu Fujisawa and Kosei Matsue

Figure S1

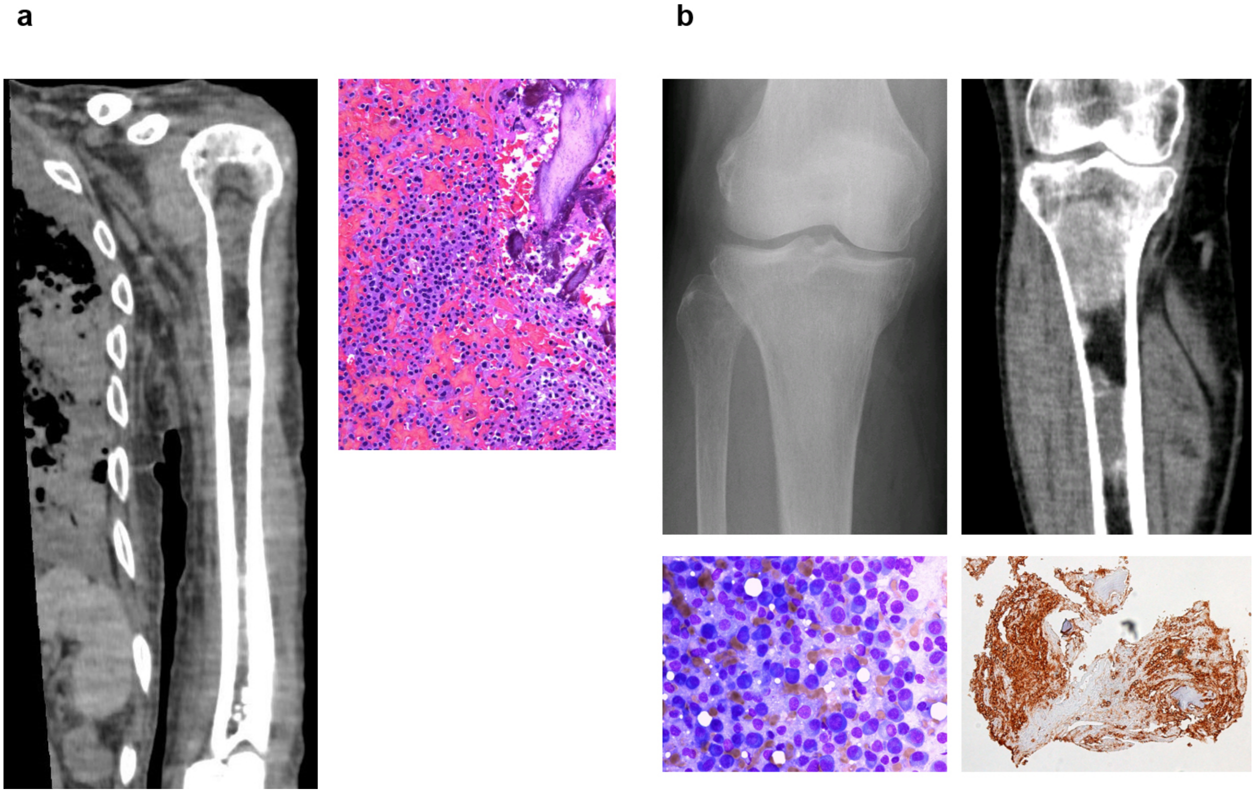

Figure S1. Two cases with advanced symptomatic multiple myeloma with bone marrow infiltrations detected by MDCT as well as histological diagnosis. (**a**) A postmortem coronal CT image with hyperattenuating lesions in the middle of the left humerus (left panel). Necropsy in this lesion revealed massive infiltration of malignant plasma cells (right panel) (Haematoxylin and eosin staining). (**b**) A patient presenting with oedema and a dull pain in the right lower leg. A plain radiograph did not show significant lytic lesions (upper left), but MDCT detected hyperattenuating lesions (upper right). A bone marrow smear (lower left) and biopsy (lower right) with CD138 immunohistochemical staining obtained from the right tibia showed proliferation of malignant plasma cells.

Figure S2

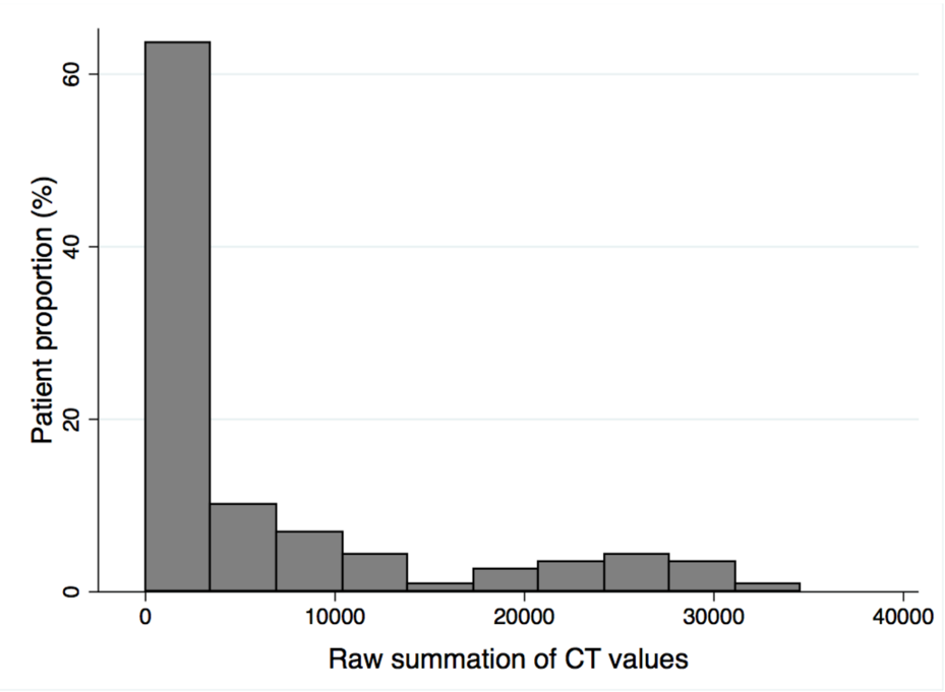

Figure S2. Histogram of raw summation of CT values in patients with symptomatic multiple myeloma.

Figure S3

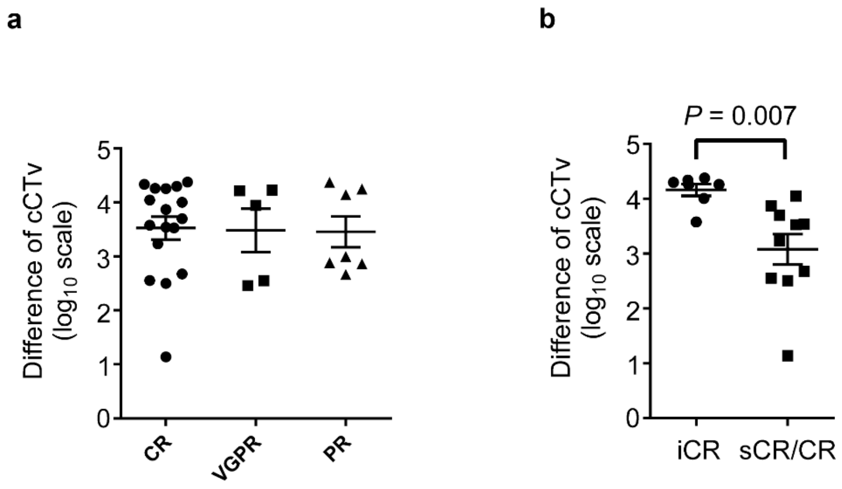

Figure S3. (**a**) Comparison of differences in cCTv calculated from initial and follow-up MDCT images after treatment, with treatment responses defined by the International Myeloma Working Group. cCTv, Cumulative CT value; CR, complete response; VGPR, very good partial response; PR, partial response. (**b**) Comparison of differences in cCTv in patients who achieved CR. iCR, immunophenotypic CR; sCR, stringent CR.

Supplemental table

Characteristics of patients with cumulative CT values (cCTv) above and below the median

Parameters	Low cCTv (N = 45 (%)	High cCTv (N = 46) (%)	*P* value
**Age (mean)**	74.2	70.4	0.079*
**Male sex**	24 (53)	25 (54)	0.923
**IgG subtype**	27 (60)	23 (50)	0.338
**D-S stage III**	24 (53)	39 (85)	0.001
**ISS III**	15 (33)	28 (61)	0.009
**High-risk CA**	6 (13)	13 (28)	0.080
**High LDH**	4 (9)	10 (22)	0.089
**Revised ISS III**	4 (9)	17 (37)	0.001
**HDM + ASCT**	11 (24)	19 (41)	0.087

Abbreviations: avg, average; IgG, Immunoglobulin G; D-S, Durie-Salmon; ISS, International Staging System; CA, chromosomal abnormalities; LDH, lactate dehydrogenase; HDM, high-dose melphalan; ASCT, autologous stem cell transplantation

**P* value was provided by Mann-Whitney U test. All other *P* values were provided by chi-square tests.
